# First documented case of avian influenza (H5N1) virus infection in a lion

**DOI:** 10.1038/emi.2016.127

**Published:** 2016-12-21

**Authors:** Quanjiao Chen, Hanzhong Wang, Lihua Zhao, Liping Ma, Runkun Wang, Yongsong Lei, Yong Li, Guoxiang Yang, Jing Chen, Guang Chen, Liqiang Li, Tao Jin, Jiandong Li, Xin Liu, Xun Xu, Gary Wong, Lei Liu, Yingxia Liu, Weifeng Shi, Yuhai Bi, George F Gao

**Affiliations:** 1 CAS Key Laboratory of Special Pathogens and Biosafety, Wuhan Institute of Virology, Chinese Academy of Sciences, Wuhan 430071, Hubei Province, China; 2 Center for Influenza Research and Early-Warning (CASCIRE), Chinese Academy of Sciences, Beijing 100101, China; 3 Graduate School, University of Chinese Academy of Sciences, Beijing 100049, China; 4 The Monitoring Center of Wildlife Diseases and Resource of Hubei Province, Wuhan 430075, Hubei Province, China; 5 BGI-Shenzhen, Shenzhen 518083, Guangdong Province, China; 6 CAS Key Laboratory of Pathogenic Microbiology and Immunology, Institute of Microbiology, Chinese Academy of Sciences, Beijing 100101, China; 7 Shenzhen Key Laboratory of Pathogen and Immunity, State Key Discipline of Infectious Disease, Shenzhen Third People's Hospital, Shenzhen 518040, Guangdong Province, China; 8 Institute of Pathogen Biology, Taishan Medical College, Taian 27100, Shandong Province, China

**Dear Editor,**

On 12 April 2016, a 3-year-old male lion died in Ezhou zoo, Hubei province, China. Clinical symptoms include droopiness and anorexia several days before death, and no improvement was observed after symptomatic treatment. The body temperature of the lion, measured the night before its death, was 38.8 °C–38.9 °C. Necropsy findings included severe congestion and edema in the lungs, slight intestinal bloating and red residual urine in the bladder. Histopathological changes consisted of renal tubular necrosis and interstitial hemorrhage in the kidneys. In addition, there were pulmonary congestion, inflammatory infiltration and interstitial proliferation in the lungs, consistent with respiratory failure (Figures 1A and 1B).

No bacteria were detected in the tissues. The spleen, kidney, liver, lung, blood and urine were collected for detection of several viral pathogens using PCR or reverse transcription-PCR in which the specimens were shown to be negative for feline immunodeficiency virus, canine parvovirus, canine and feline distemper virus, and avian paramyxovirus. However, the samples tested positive for H5N1 avian influenza virus (AIV). Therefore, the supernatant of the homogenized tissues were inoculated into 10-day-old specific pathogen-free embryonated chicken eggs for virus isolation. Live virus was isolated from the lung, kidney and blood samples, and designated as A/Lion/Hubei/1-2F/2016, A/Lion/Hubei/1-2S/2016 and A/Lion/Hubei/1-2X/2016, respectively. These samples were sent for next-generation sequencing (NGS). There were no differences in the nucleotide sequences from the whole genomes of these viruses. A/Lion/Hubei/1-2F/2016 (designated 1-2F in this manuscript) was then selected for subsequent analyses, and the nucleotide sequences for all eight segments of this strain have been deposited at GenBank, under accession numbers KX571056-KX571063.

Phylogenetic analysis of the HA (Figure 1C), NA (Supplementary Figure S1B), NP (Supplementary Figure S1F) and NS (Supplementary Figure S1H) genes of the 1-2F isolate showed that they belonged to viruses from clade 2.3.2.1c H5N1.^1^ The M and PB2 segments were found to be closely related to several H5N1 strains in China, which originated from an H9N2 lineage circulating in China and Southeast Asia (Supplementary Figures S1G and S1C). The PA and PB1 genes fell within a small H5N1 clade, which comes from a low pathogenic AIV gene pool existing mostly in waterfowl and wild birds (Supplementary Figures S1E and S1D). Therefore, the 1-2F isolate is a reassortant virus. Viruses belonging to this genotype were isolated from different provinces (Zhejiang, Yunnan and Hubei) in China, from different hosts (chickens, ducks, peacocks and tigers) and from different years (2014–2016), suggesting that the distribution of this H5N1 genotype is wider than previously thought.

In particular, all eight genes in 1-2F were similar to and clustered with those of a clade 2.3.2.1 H5N1 virus isolated from a tiger (A/tiger/Yunnan/tig1508/2015(H5N1)) that had died during August 2015.^2^ Since clade 2.3.2.1 viruses are prevalent among poultry in China and have been repeatedly detected in wild birds,^3, 4, 5^ more infections with clade 2.3.2.1 viruses in mammals can be expected.

To investigate the source of the infection, environmental samples taken from the lion and the birds in the zoo were collected for NGS analysis (Supplementary Technical Appendix). The sequencing depth was 2G per sample. H5 and N1 were not found in the environmental samples collected from birds; however, 6689 H5 and 5936 N1 reads were detected in the drinking water of the lion. These results suggested that the lion may not have acquired the H5N1 infection from the birds in the zoo. Alternatively, the lion was fed with frozen chicken carcasses, and therefore the most plausible source of the infection might be chickens, which may have been contaminated by H5N1 AIV.

Genetic analysis further showed that the HA gene of 1-2F contained multiple basic amino acids (RERRRKR) at the cleavage site. The amino-acid residues in the receptor-binding sites of HA exhibited avian characteristics (226-228 QSG). Furthermore, E627 and D701 residues in the PB2 protein suggested that the virus had not yet adapted to the mammalian host.^6, 7^ A 20 amino-acid deletion of NA was detected at positions 49–68, which may increase virus adaptation to domestic fowl and virulence in mammals.^8^ The H274 residue of NA and N31 in M2 suggested that the virus was sensitive to oseltamivir while resistant to amantadine. There were no differences in the key sites between the H5N1 AIV isolated from lions and chickens (A/chicken/Wuhan/HAQL07/2014(H5N1)), and only a difference of 1–2 amino acids when compared with viruses derived from tigers and wild birds, respectively (Supplementary Table S1).

In summary, we documented the first infection of a lion with a highly pathogenic H5N1 AIV, expanding the host range of AIV. This virus was a reassortant from circulating H5N1/H9N2 viruses and remained prevalent among poultry. Since this virus was highly virulent to the lion and that AIV has been reported to cause deaths in tigers and leopards,^9^ we believe that captive animals are under certain risk of AIV infections, and increased surveillance is needed to track and chart the ongoing evolution of circulating AIV with respect to their potential to infect susceptible mammalian hosts.

## Figures and Tables

**Figure 1:**
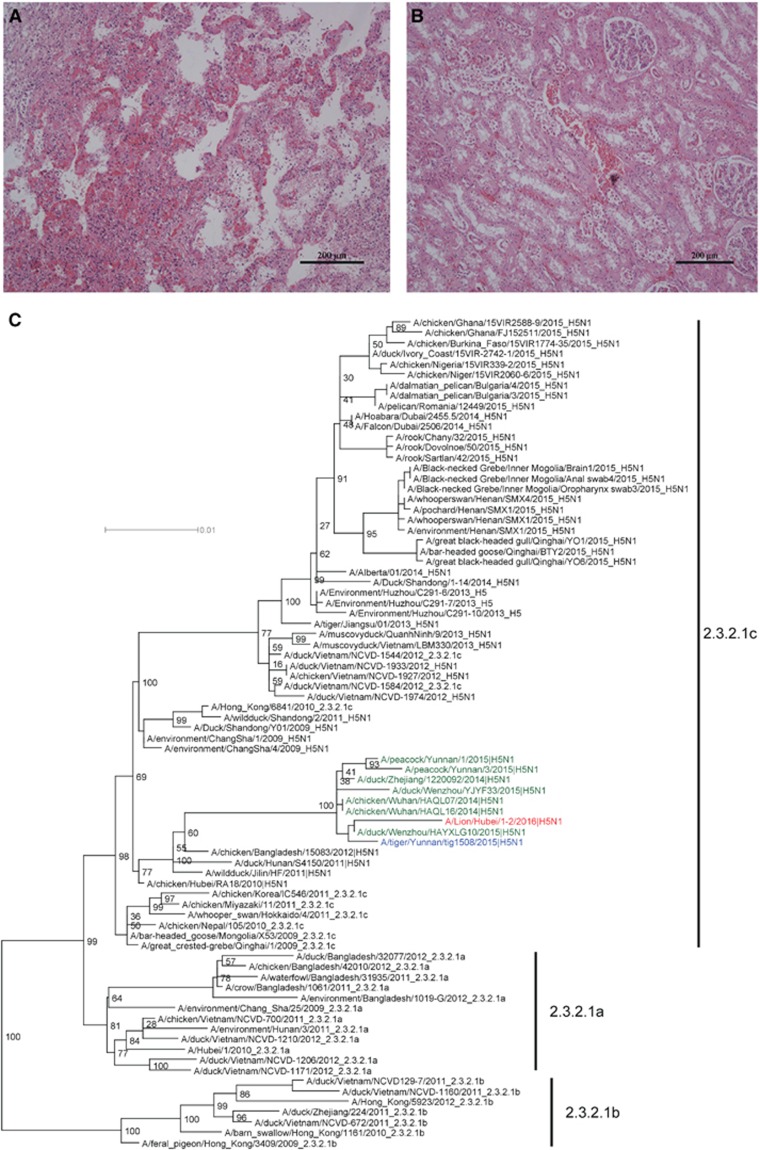
Histopathological changes (**A** Lung, **B** kidney, magnification × 200) in the dead lion and phylogenetic analysis of the H5 genes (**C**).

## Supplementary Material

Supplementary Table S1

Supplementary Technical Appendix
