# Supplementary material for: First documented case of avian influenza (H5N1) virus infection in a lion
Source: Emerg Microbes Infect. 2016 Dec 21;5(12):e125–. doi: 10.1038/emi.2016.127 (PMC5180371; doi:10.1038/emi.2016.127)
Supplement: Supplementary Figure S1 [file emi2016127x1.pdf]

A-H5

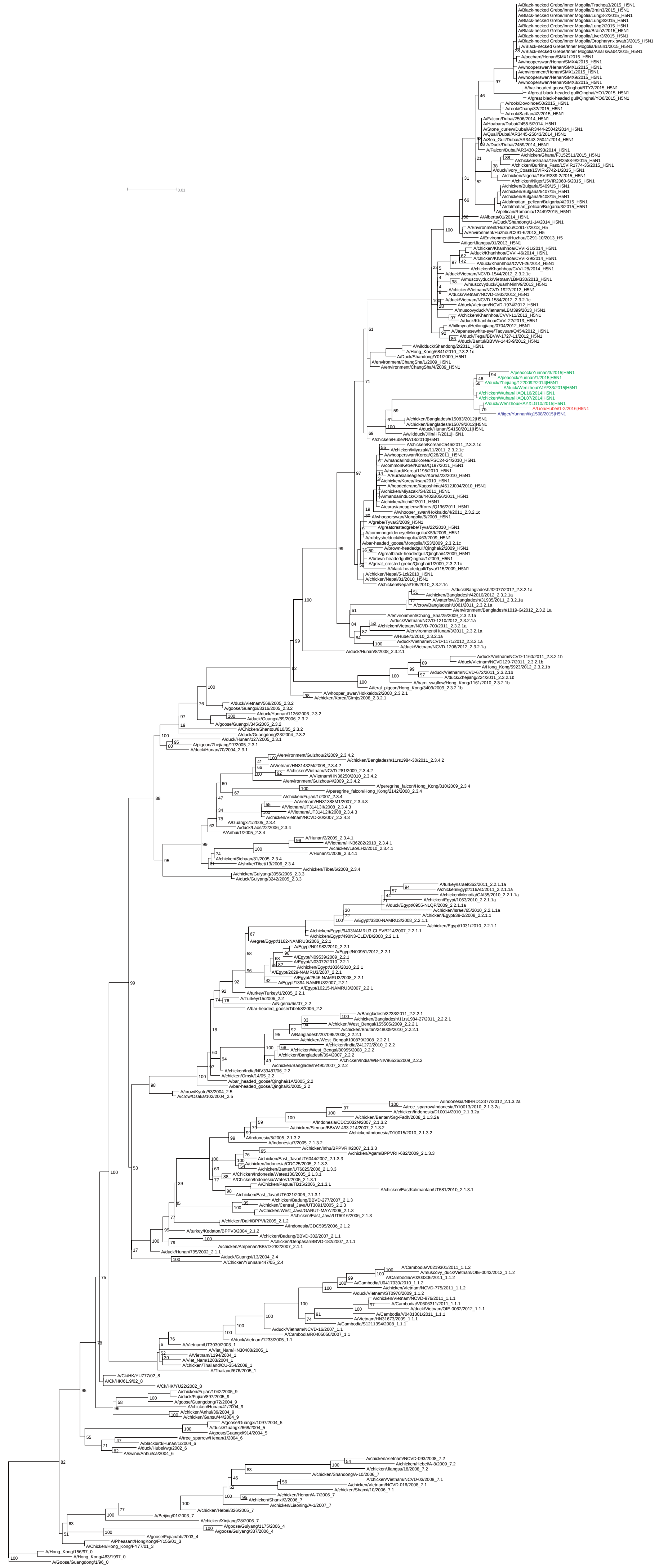

B-N1

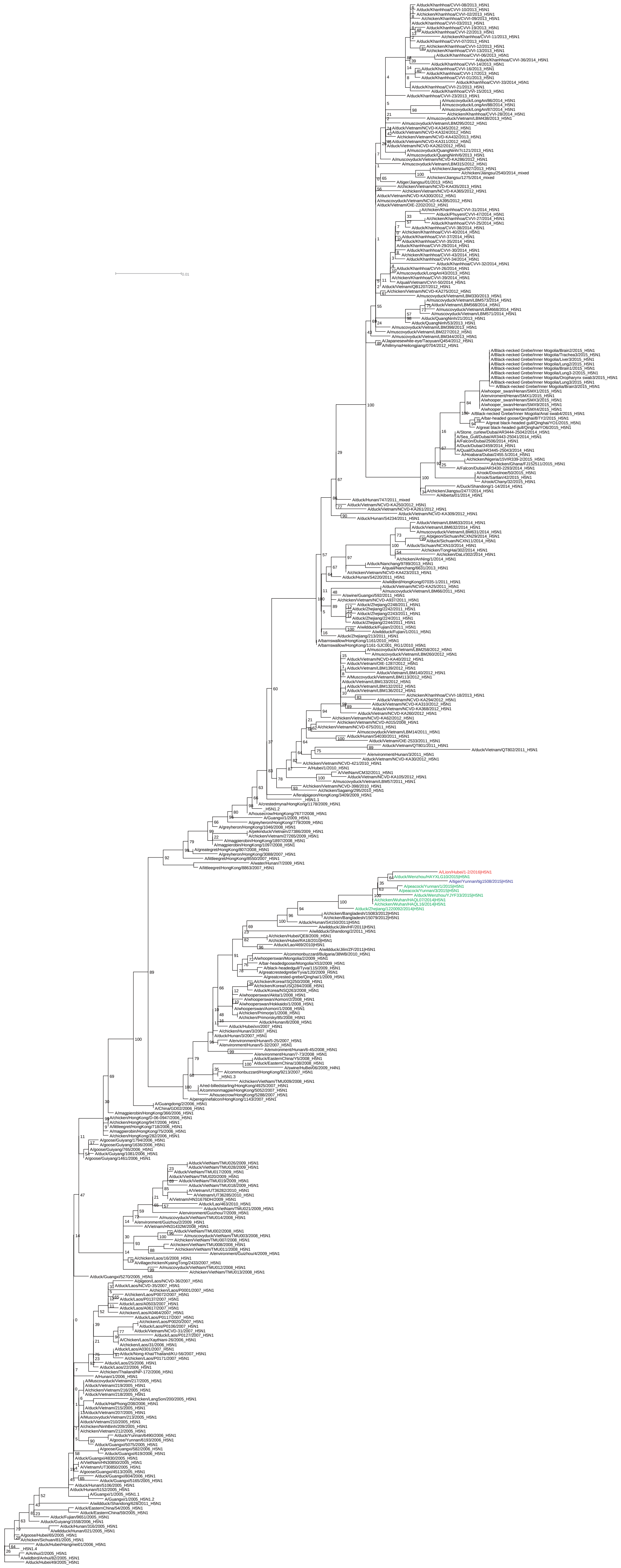

C-PB2

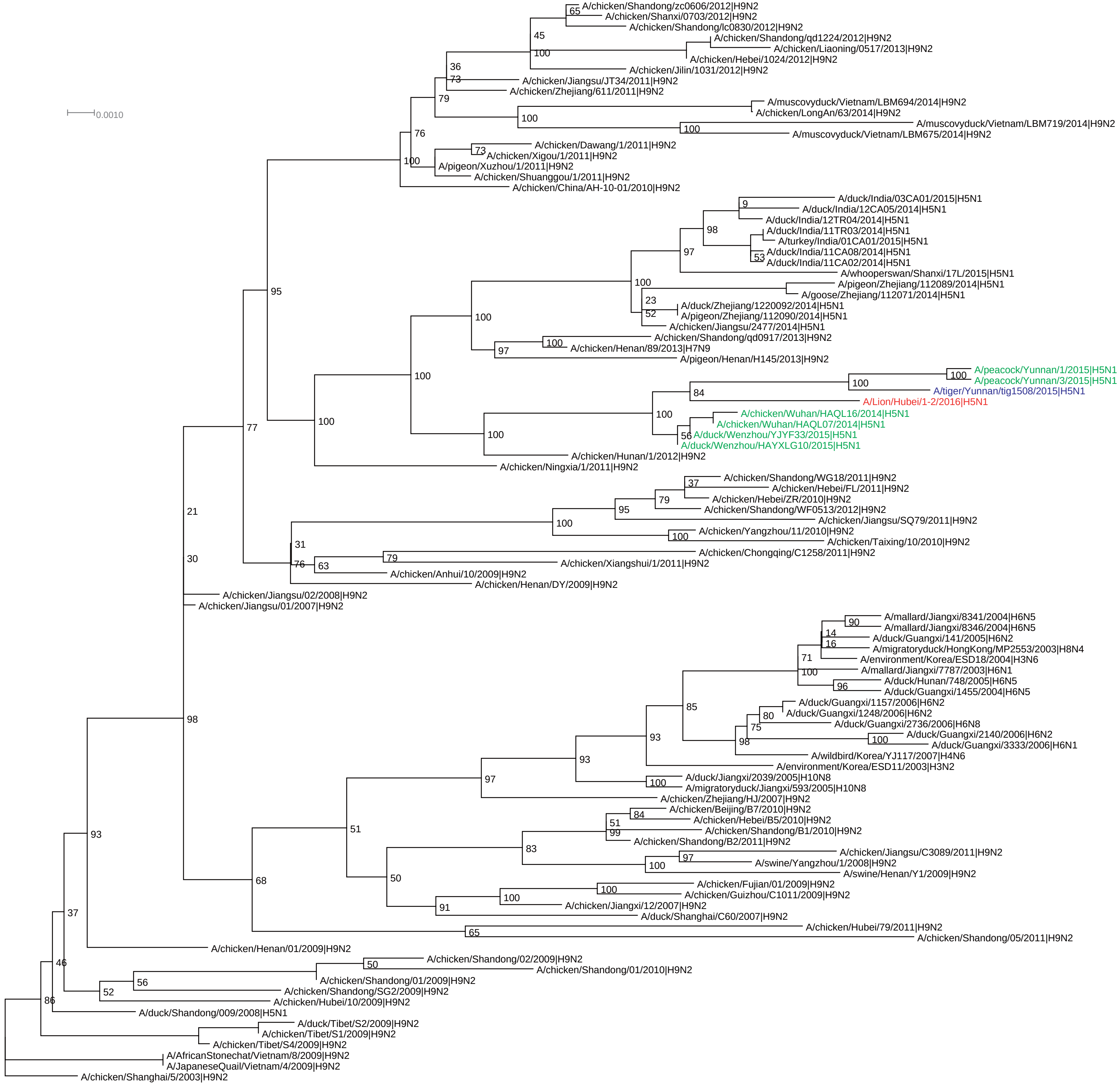

0.0010
A/chicken/Shandong/zc0606/2012|H9N2
A/chicken/Shanxi/0703/2012|H9N2
A/chicken/Shandong/lc0830/2012|H9N2
A/chicken/Shandong/qd1224/2012|H9N2
A/chicken/Liaoning/0517/2013|H9N2
A/chicken/Hebei/1024/2012|H9N2
A/chicken/Jilin/1031/2012|H9N2
A/chicken/Jiangsu/JT34/2011|H9N2
A/chicken/Zhejiang/611/2011|H9N2
A/muscovyduck/Vietnam/LBM694/2014|H9N2
A/chicken/LongAn/63/2014|H9N2
A/muscovyduck/Vietnam/LBM719/2014|H9N2
A/muscovyduck/Vietnam/LBM675/2014|H9N2
A/chicken/Dawang/1/2011|H9N2
A/chicken/Xigou/1/2011|H9N2
A/pigeon/Xuzhou/1/2011|H9N2
A/chicken/Shuanggou/1/2011|H9N2
A/chicken/China/AH-10-01/2010|H9N2
A/duck/India/03CA01/2015|H5N1
A/duck/India/12CA05/2014|H5N1
A/duck/India/12TR04/2014|H5N1
A/duck/India/11TR03/2014|H5N1
A/turkey/India/01CA01/2015|H5N1
A/duck/India/11CA08/2014|H5N1
A/duck/India/11CA02/2014|H5N1
A/whooperswan/Shanxi/17L/2015|H5N1
A/pigeon/Zhejiang/112089/2014|H5N1
A/goose/Zhejiang/112071/2014|H5N1
A/duck/Zhejiang/1220092/2014|H5N1
A/pigeon/Zhejiang/112090/2014|H5N1
A/chicken/Jiangsu/2477/2014|H5N1
A/chicken/Shandong/qd0917/2013|H9N2
A/chicken/Henan/89/2013|H7N9
A/pigeon/Henan/H145/2013|H9N2
A/peacock/Yunnan/1/2015|H5N1
A/peacock/Yunnan/3/2015|H5N1
A/tiger/Yunnan/tig1508/2015|H5N1
A/Lion/Hubei/1-2/2016|H5N1
A/chicken/Wuhan/HAQL16/2014|H5N1
A/chicken/Wuhan/HAQL07/2014|H5N1
A/duck/Wenzhou/YJYF33/2015|H5N1
A/duck/Wenzhou/HAYXLG10/2015|H5N1
A/chicken/Hunan/1/2012|H9N2
A/chicken/Ningxia/1/2011|H9N2
A/chicken/Shandong/WG18/2011|H9N2
A/chicken/Hebei/FL/2011|H9N2
A/chicken/Hebei/ZR/2010|H9N2
A/chicken/Shandong/WF0513/2012|H9N2
A/chicken/Jiangsu/SQ79/2011|H9N2
A/chicken/Yangzhou/11/2010|H9N2
A/chicken/Taixing/10/2010|H9N2
A/chicken/Chongqing/C1258/2011|H9N2
A/chicken/Xiangshui/1/2011|H9N2
A/chicken/Anhui/10/2009|H9N2
A/chicken/Henan/DY/2009|H9N2
A/chicken/Jiangsu/02/2008|H9N2
A/chicken/Jiangsu/01/2007|H9N2
A/mallard/Jiangxi/8341/2004|H6N5
A/mallard/Jiangxi/8346/2004|H6N5
A/duck/Guangxi/141/2005|H6N2
A/migratoryduck/HongKong/MP2553/2003|H8N4
A/environment/Korea/ESD18/2004|H3N6
A/mallard/Jiangxi/7787/2003|H6N1
A/duck/Hunan/748/2005|H6N5
A/duck/Guangxi/1455/2004|H6N5
A/duck/Guangxi/1157/2006|H6N2
A/duck/Guangxi/1248/2006|H6N2
A/duck/Guangxi/2736/2006|H6N8
A/duck/Guangxi/2140/2006|H6N2
A/duck/Guangxi/3333/2006|H6N1
A/wildbird/Korea/YJ117/2007|H4N6
A/environment/Korea/ESD11/2003|H3N2
A/duck/Jiangxi/2039/2005|H10N8
A/migratoryduck/Jiangxi/593/2005|H10N8
A/chicken/Zhejiang/HJ/2007|H9N2
A/chicken/Beijing/B7/2010|H9N2
A/chicken/Hebei/B5/2010|H9N2
A/chicken/Shandong/B1/2010|H9N2
A/chicken/Shandong/B2/2011|H9N2
A/chicken/Jiangsu/C3089/2011|H9N2
A/swine/Yangzhou/1/2008|H9N2
A/swine/Henan/Y1/2009|H9N2
A/chicken/Fujian/01/2009|H9N2
A/chicken/Guizhou/C1011/2009|H9N2
A/chicken/Jiangxi/12/2007|H9N2
A/duck/Shanghai/C60/2007|H9N2
A/chicken/Hubei/79/2011|H9N2
A/chicken/Shandong/05/2011|H9N2
A/chicken/Henan/01/2009|H9N2
A/chicken/Shandong/02/2009|H9N2
A/chicken/Shandong/01/2010|H9N2
A/chicken/Shandong/01/2009|H9N2
A/chicken/Shandong/SG2/2009|H9N2
A/chicken/Hubei/10/2009|H9N2
A/duck/Shandong/009/2008|H5N1
A/duck/Tibet/S2/2009|H9N2
A/chicken/Tibet/S1/2009|H9N2
A/chicken/Tibet/S4/2009|H9N2
A/AfricanStonechat/Vietnam/8/2009|H9N2
A/JapaneseQuail/Vietnam/4/2009|H9N2
A/chicken/Shanghai/5/2003|H9N2

D-PB1

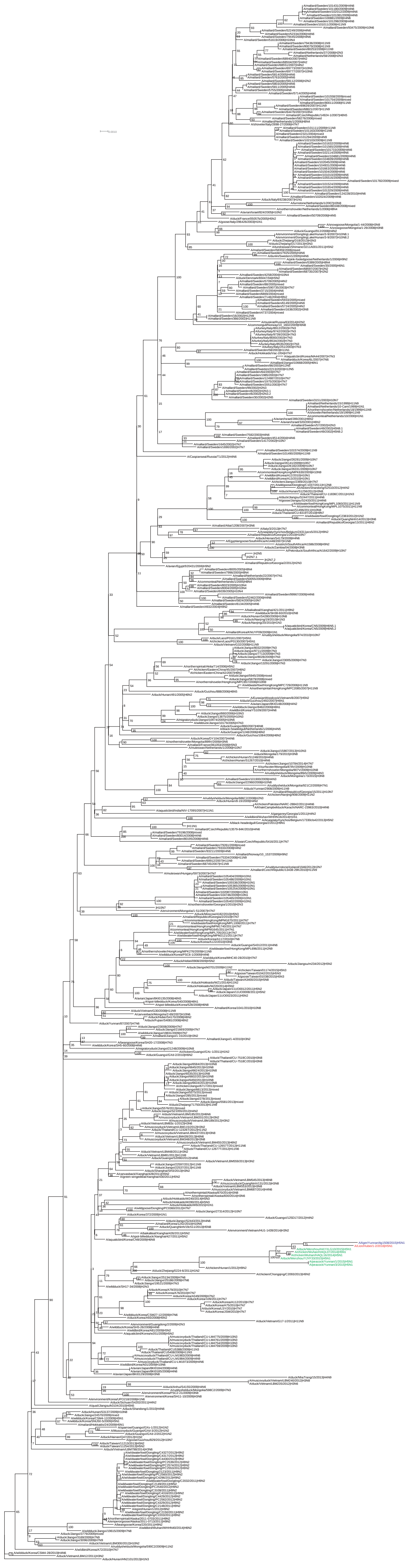

E-PA

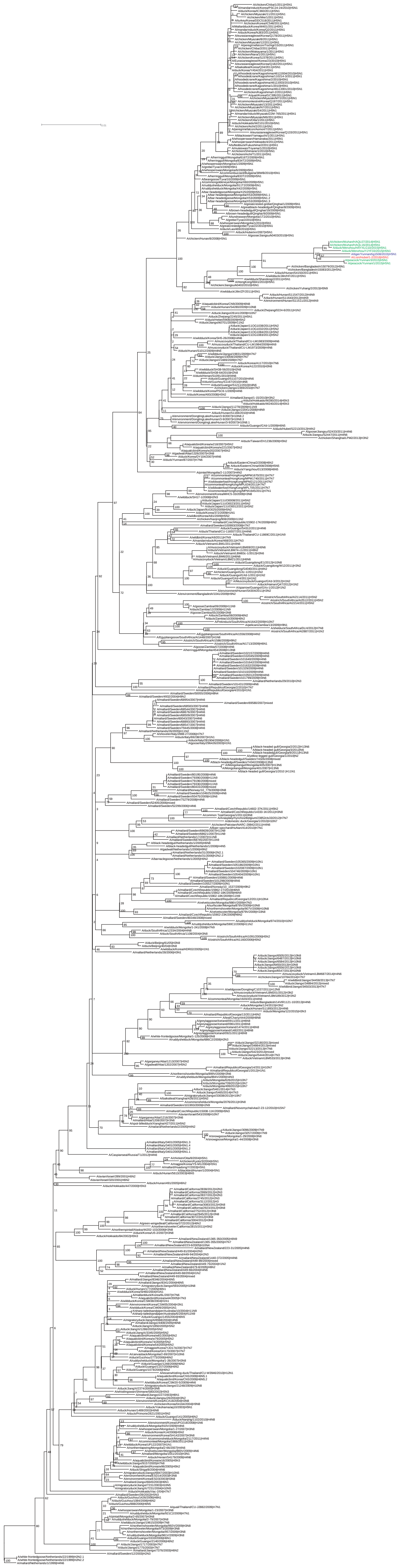

F-NP

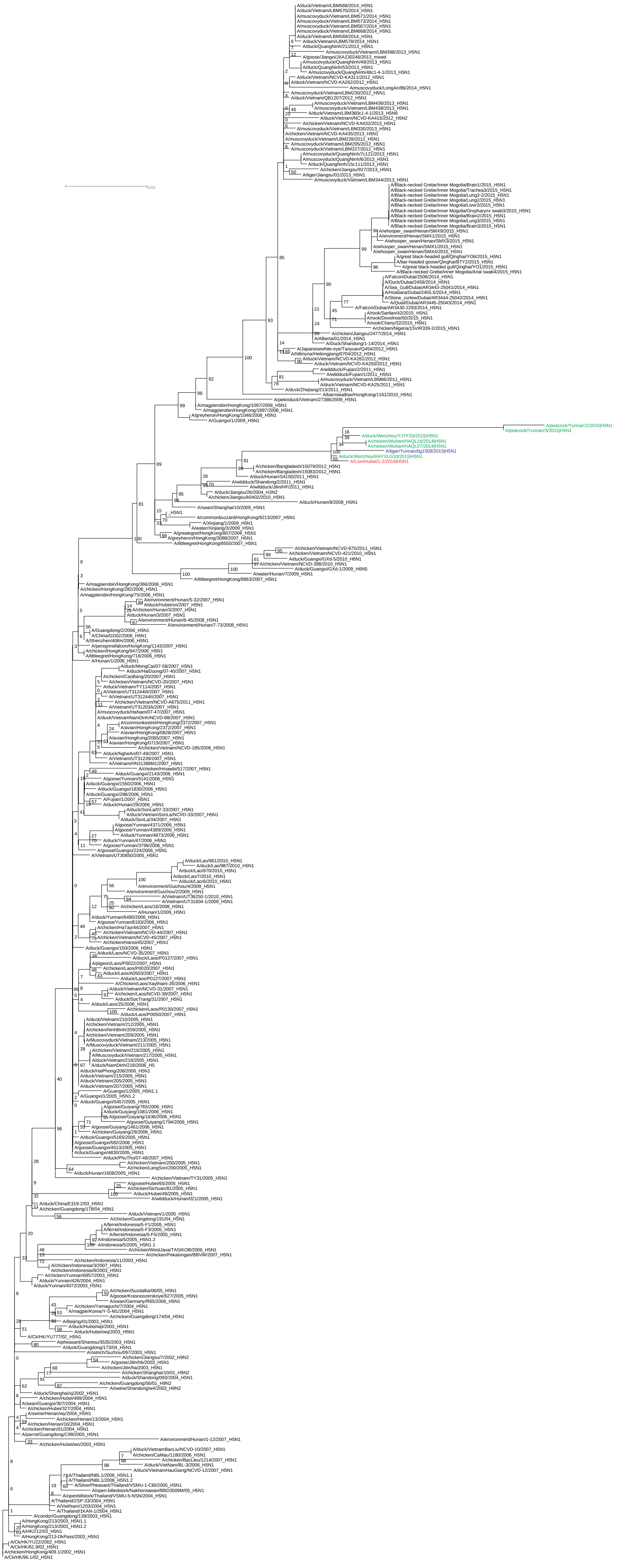

G-M

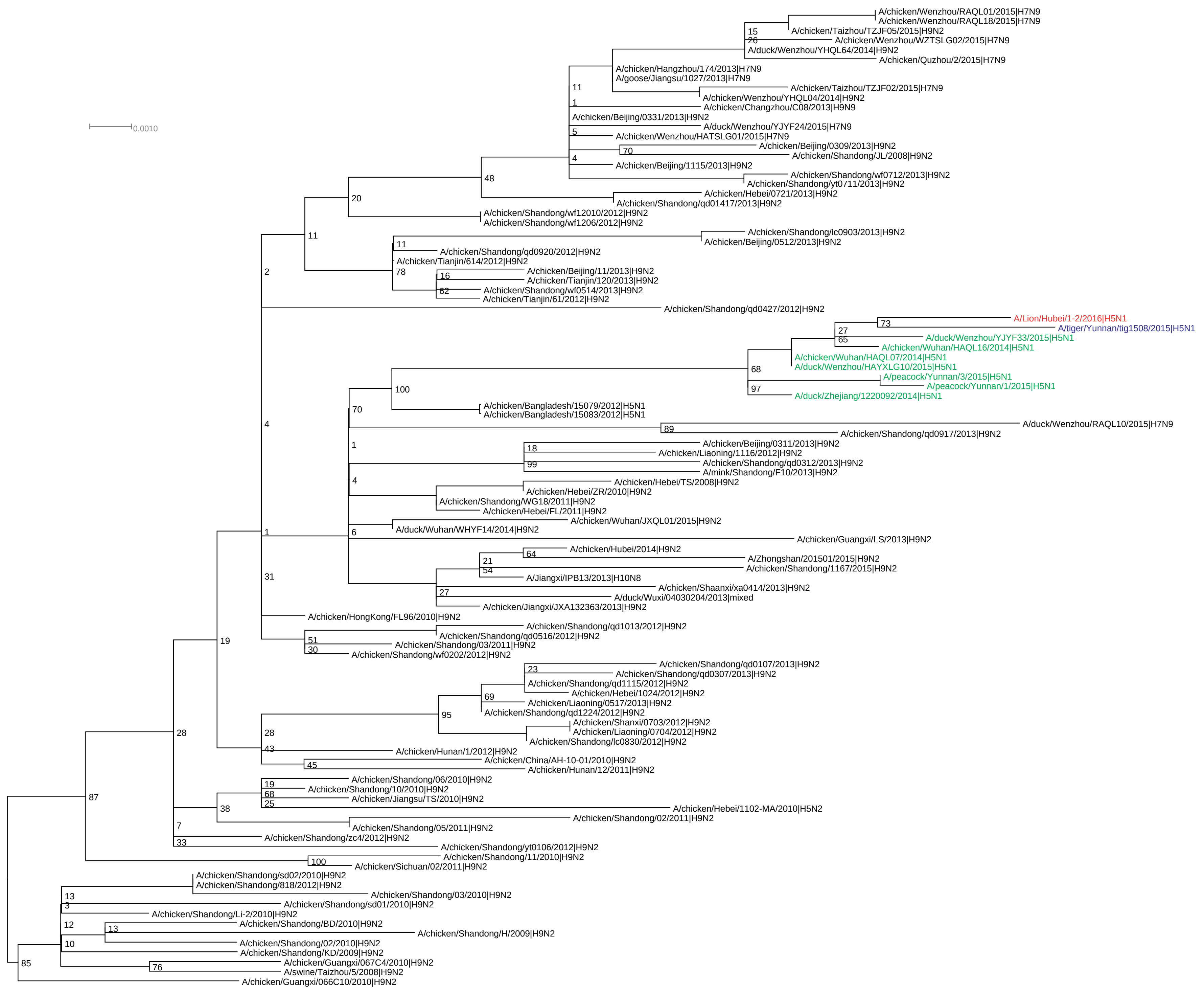
0.0010
A/chicken/Wenzhou/RAQL01/2015|H7N9
A/chicken/Wenzhou/RAQL18/2015|H7N9
A/chicken/Taizhou/TZJF05/2015|H9N2
A/chicken/Wenzhou/WZTSLG02/2015|H7N9
A/duck/Wenzhou/YHQL64/2014|H9N2
A/chicken/Quzhou/2/2015|H7N9
A/chicken/Hangzhou/174/2013|H7N9
A/goose/Jiangsu/1027/2013|H7N9
A/chicken/Taizhou/TZJF02/2015|H7N9
A/chicken/Wenzhou/YHQL04/2014|H9N2
A/chicken/Changzhou/C08/2013|H9N9
A/chicken/Beijing/0331/2013|H9N2
A/duck/Wenzhou/YJYF24/2015|H7N9
A/chicken/Wenzhou/HATSLG01/2015|H7N9
A/chicken/Beijing/0309/2013|H9N2
A/chicken/Shandong/JL/2008|H9N2
A/chicken/Beijing/1115/2013|H9N2
A/chicken/Shandong/wf0712/2013|H9N2
A/chicken/Shandong/yt0711/2013|H9N2
A/chicken/Hebei/0721/2013|H9N2
A/chicken/Shandong/qd01417/2013|H9N2
A/chicken/Shandong/wf12010/2012|H9N2
A/chicken/Shandong/wf1206/2012|H9N2
A/chicken/Shandong/lc0903/2013|H9N2
A/chicken/Beijing/0512/2013|H9N2
A/chicken/Shandong/qd0920/2012|H9N2
A/chicken/Tianjin/614/2012|H9N2
A/chicken/Beijing/11/2013|H9N2
A/chicken/Tianjin/120/2013|H9N2
A/chicken/Shandong/wf0514/2013|H9N2
A/chicken/Tianjin/61/2012|H9N2
A/chicken/Shandong/qd0427/2012|H9N2
A/Lion/Hubei/1-2/2016|H5N1
A/tiger/Yunnan/tig1508/2015|H5N1
A/duck/Wenzhou/YJYF33/2015|H5N1
A/chicken/Wuhan/HAQL16/2014|H5N1
A/chicken/Wuhan/HAQL07/2014|H5N1
A/duck/Wenzhou/HAYXLG10/2015|H5N1
A/peacock/Yunnan/3/2015|H5N1
A/peacock/Yunnan/1/2015|H5N1
A/duck/Zhejiang/1220092/2014|H5N1
A/chicken/Bangladesh/15079/2012|H5N1
A/chicken/Bangladesh/15083/2012|H5N1
A/duck/Wenzhou/RAQL10/2015|H7N9
A/chicken/Shandong/qd0917/2013|H9N2
A/chicken/Beijing/0311/2013|H9N2
A/chicken/Liaoning/1116/2012|H9N2
A/chicken/Shandong/qd0312/2013|H9N2
A/mink/Shandong/F10/2013|H9N2
A/chicken/Hebei/TS/2008|H9N2
A/chicken/Hebei/ZR/2010|H9N2
A/chicken/Shandong/WG18/2011|H9N2
A/chicken/Hebei/FL/2011|H9N2
A/chicken/Wuhan/JXQL01/2015|H9N2
A/duck/Wuhan/WHYF14/2014|H9N2
A/chicken/Guangxi/LS/2013|H9N2
A/chicken/Hubei/2014|H9N2
A/Zhongshan/201501/2015|H9N2
A/chicken/Shandong/1167/2015|H9N2
A/Jiangxi/IPB13/2013|H10N8
A/chicken/Shaanxi/xa0414/2013|H9N2
A/duck/Wuxi/04030204/2013|mixed
A/chicken/Jiangxi/JXA132363/2013|H9N2
A/chicken/HongKong/FL96/2010|H9N2
A/chicken/Shandong/qd1013/2012|H9N2
A/chicken/Shandong/qd0516/2012|H9N2
A/chicken/Shandong/03/2011|H9N2
A/chicken/Shandong/wf0202/2012|H9N2
A/chicken/Shandong/qd0107/2013|H9N2
A/chicken/Shandong/qd0307/2013|H9N2
A/chicken/Shandong/qd1115/2012|H9N2
A/chicken/Hebei/1024/2012|H9N2
A/chicken/Liaoning/0517/2013|H9N2
A/chicken/Shandong/qd1224/2012|H9N2
A/chicken/Shanxi/0703/2012|H9N2
A/chicken/Liaoning/0704/2012|H9N2
A/chicken/Shandong/lc0830/2012|H9N2
A/chicken/Hunan/1/2012|H9N2
A/chicken/China/AH-10-01/2010|H9N2
A/chicken/Hunan/12/2011|H9N2
A/chicken/Shandong/06/2010|H9N2
A/chicken/Shandong/10/2010|H9N2
A/chicken/Jiangsu/TS/2010|H9N2
A/chicken/Hebei/1102-MA/2010|H5N2
A/chicken/Shandong/02/2011|H9N2
A/chicken/Shandong/05/2011|H9N2
A/chicken/Shandong/zc4/2012|H9N2
A/chicken/Shandong/yt0106/2012|H9N2
A/chicken/Shandong/11/2010|H9N2
A/chicken/Sichuan/02/2011|H9N2
A/chicken/Shandong/sd02/2010|H9N2
A/chicken/Shandong/818/2012|H9N2
A/chicken/Shandong/03/2010|H9N2
A/chicken/Shandong/sd01/2010|H9N2
A/chicken/Shandong/Li-2/2010|H9N2
A/chicken/Shandong/BD/2010|H9N2
A/chicken/Shandong/H/2009|H9N2
A/chicken/Shandong/02/2010|H9N2
A/chicken/Shandong/KD/2009|H9N2
A/chicken/Guangxi/067C4/2010|H9N2
A/swine/Taizhou/5/2008|H9N2
A/chicken/Guangxi/066C10/2010|H9N2

H-NS

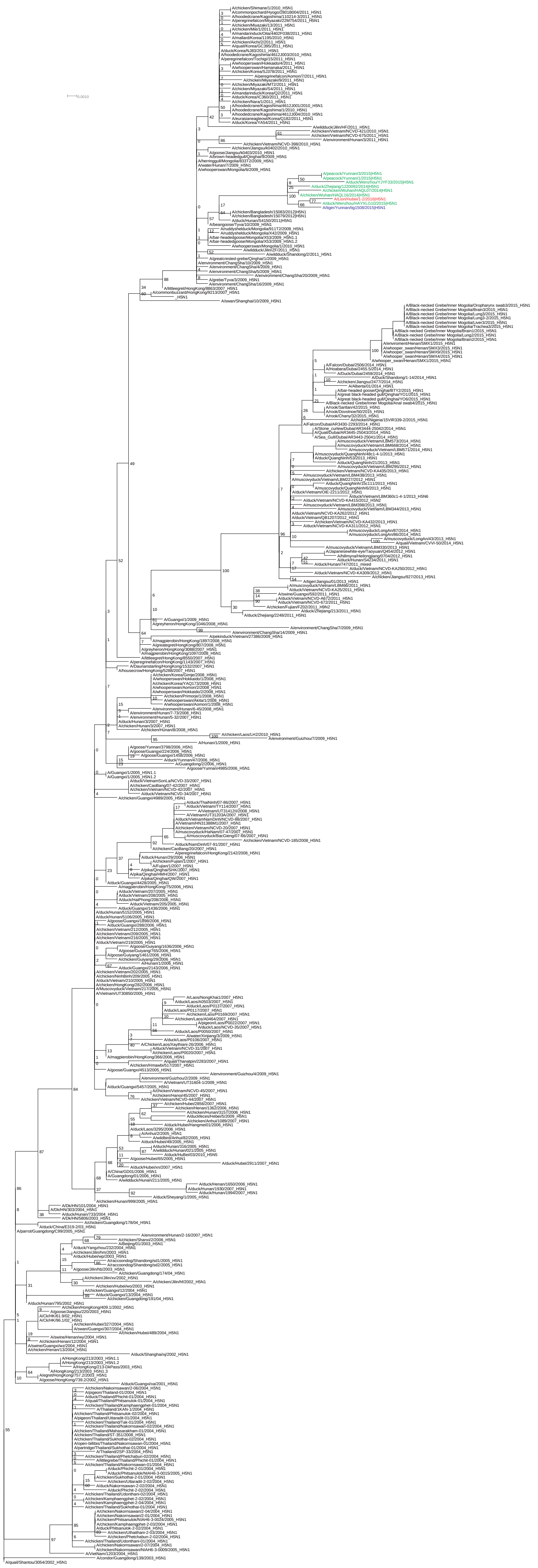

Supplementary Figure S1 Phylogenetic analysis of the H5 (**A**), N1 (**B**), PB2 (**C**), PB1 (**D**), PA (**E**), NP (**F**), M (**G**) and NS (**H**) genes of the H5N1 isolates. The maximum-likelihood phylogenetic trees were inferred by the software RAxML under the GAMMAGTR model with 1000 bootstraps.
