# Supplementary material for: First documented case of avian influenza (H5N1) virus infection in a lion
Source: Emerg Microbes Infect. 2016 Dec 21;5(12):e125–. doi: 10.1038/emi.2016.127 (PMC5180371; doi:10.1038/emi.2016.127)
Supplement: Supplementary Table S1 [file emi2016127x2.doc]

**Supplementary Table** S1 Molecular characterizations of H5N1 viruses.

| **Viruses (H5N1) ^†^** | **Collection**  **date** | **HA (****H3 numbering)** | | | | | |  | **PB2** | | | |  | **NA** | |  | **M2** | | | |  | **PB1-F2** |
| --- | --- | --- | --- | --- | --- | --- | --- | --- | --- | --- | --- | --- | --- | --- | --- | --- | --- | --- | --- | --- | --- | --- |
|  |  | Connecting  peptide | 160 | 224 | 226 | 228 | 318 |  | 590 | 591 | 627 | 701 |  | 274* | Stalk deletion |  | 26 | 27 | 30 | 31 |  | 58-90 truncated |
| Lion | 2016 | RERRRKR | T | N | Q | G | T |  | G | Q | E | D |  | H | 49-68 |  | L | G | A | N |  | NO |
| Tiger | 2015 | RERRRKR | T | N | Q | G | T |  | G | Q | E | N |  | H | 49-68 |  | L | G | A | N |  | NO |
| Chicken | 2014 | RERRRKR | T | N | Q | G | T |  | G | Q | E | D |  | H | 49-68 |  | L | G | A | N |  | NO |
| Wild birds | 2009 | RERRRKR | A | N | Q | G | T |  | G | Q | E | D |  | H | 49-68 |  | L | V | A | S |  | NO |

* N2 numbering

^†^Lion: A/Lion/Hubei/1-2F/2016(H5N1); Tiger: A/tiger/Yunnan/tig1508/2015(H5N1);

Chicken: A/chicken/Wuhan/HAQL07/2014(H5N1); Wild birds: A/great_crested-grebe/Qinghai/1/2009(H5N1)
