# Supplementary material for: First documented case of avian influenza (H5N1) virus infection in a lion
Source: Emerg Microbes Infect. 2016 Dec 21;5(12):e125–. doi: 10.1038/emi.2016.127 (PMC5180371; doi:10.1038/emi.2016.127)
Supplement: Supplementary Technical Appendix [file emi2016127x3.doc]

**Supplementary Technical Appendix**

**Virus Isolation and Sequencing**

A three-year old male lion died on April 12^th^ 2016 in Ezhou Zoo, Hubei Province. The spleen, kidney, liver, lung, blood in heart and urine specimens were obtained and homogenized with PBS containing 0.1% BSA. The tissue homogenate was inoculated into the allantoic cavities of 10-day-old specific pathogen-free (SPF) embryonated chicken eggs for 48 to 72 hours at 37°C. Viral RNA from the allantoic fluid was extracted by MiniBEST Universal RNA Extraction Kit and RNA was reverse-transcribed and amplified with the PrimeScript™ One Step RT-PCR Kit according to manufacturer instructions (Takara), using influenza A-specific primers.^1-4^

[Next generation sequencing](http://www.baidu.com/link?url=sIsUdFF2Tb2ahVrc11zJnkyhh3HfmkJ3IMmkqd7lbr3NwAGROq-S5QmSBbDBisiF-1wryXiDQhw-HO8TSrvlca) (NGS) was used to determine the whole genome sequences of the isolates and the environmental samples. The sequencing libraries were prepared by end-repairing, dA-tailing, adapter ligation and PCR amplification, according to the manufacturer instructions (Life technologies). The libraries were sequenced on an Ion Proton™ System, and sequencing depth was 0.2G for isolates and 2G for environmental samples.

Briefly, raw NGS short reads were processed by filtering out low-quality reads (8 bases with quality < 66 bp), adaptor-contaminated reads (with > 15 bp matched to the adapter sequence), poly-Ns (with 8Ns), duplication and host contaminated reads (SOAP2 (v2.21),^5^ < 5 mismatches). All reads were mapped, using TMAP v3.4.1 (https://github.com/iontorrent/TMAP) with match rate larger than 0.8, to full-length influenza A virus genomes selected from the INFLUENZA database (downloaded on August 1^st^, 2015).^6^ Best assemble reference genome segments were selected based on the coverage rate and depth of the reads. All short reads were then mapped onto the reference genome segments with match rate larger than 0.95, and the dominant base on each site was called to obtain the consensus viral genome.

**Phylogenic analysis**

Viral sequences were retrieved from GenBank after an online BLAST search. Multiple sequence alignments of each of the eight gene datasets were constructed using Muscle, respectively.^7^ Phylogenetic analyses were performed using RaxML,^8^ with the GTRGAMMA nucleotide substitution model and 200 bootstrap replicates.

**References**

1 [Gall A](http://www.ncbi.nlm.nih.gov/pubmed?term=Gall A%5BAuthor%5D&cauthor=true&cauthor_uid=18562585), [Hoffmann B](http://www.ncbi.nlm.nih.gov/pubmed?term=Hoffmann B%5BAuthor%5D&cauthor=true&cauthor_uid=18562585), [Harder T](http://www.ncbi.nlm.nih.gov/pubmed?term=Harder T%5BAuthor%5D&cauthor=true&cauthor_uid=18562585) *et al*. Universal primer set for amplification and sequencing of HA0 cleavage sites of all influenza A viruses. J Clin Microbiol. 2008; **46**: 2561-2567.

2 [Hoffmann E](http://www.ncbi.nlm.nih.gov/pubmed?term=Hoffmann E%5BAuthor%5D&cauthor=true&cauthor_uid=11811679), [Stech J](http://www.ncbi.nlm.nih.gov/pubmed?term=Stech J%5BAuthor%5D&cauthor=true&cauthor_uid=11811679), [Guan Y](http://www.ncbi.nlm.nih.gov/pubmed?term=Guan Y%5BAuthor%5D&cauthor=true&cauthor_uid=11811679) *et al*. Universal primer set for the full-length amplification of all influenza A viruses. Arch Virol. 2001; **146**: 2275-2289.

3 [Li OT](http://www.ncbi.nlm.nih.gov/pubmed?term=Li OT%5BAuthor%5D&cauthor=true&cauthor_uid=17324474), [Barr I](http://www.ncbi.nlm.nih.gov/pubmed?term=Barr I%5BAuthor%5D&cauthor=true&cauthor_uid=17324474), [Leung CY](http://www.ncbi.nlm.nih.gov/pubmed?term=Leung CY%5BAuthor%5D&cauthor=true&cauthor_uid=17324474) *et al*. Reliable universal RT-PCR assays for studying influenza polymerase subunit gene sequences from all 16 haemagglutinin subtypes. J Virol Methods 2007; **142**: 218-222.

4 Bi Y, Mei K, Shi W *et al*.Two novel reassortants of avian influenza A (H5N6) virus in China. J Gen Virol. 2015; **96**: 975-81.

5 Li, R., Yu, C., Li, Y *et al*. SOAP2: an improved ultrafast tool for short read alignment. Bioinformatics 2009; **25:** 1966-1967.

6 Bao, Y.M., Bolotov, P., Dernovoy, D *et al*. The influenza virus resource at the national center for biotechnology information. J Virol. 2008; **82:** 596-601.

7 Edgar RC. MUSCLE: multiple sequence alignment with high accuracy and high throughput. Nucleic Acids Res. 2004; **32**: 1792–1797.

8 Stamatakis A. RAxML version 8: a tool for phylogenetic analysis and post-analysis of large phylogenies. Bioinformatics 2014; **30**: 1312–1313.
